# Chinese Medicine Formula Siwu-Yin Inhibits Esophageal Precancerous Lesions by Improving Intestinal Flora and Macrophage Polarization

**DOI:** 10.3389/fphar.2022.812386

**Published:** 2022-03-03

**Authors:** Hui-Juan Shi, Xuan-Yu Chen, Xin-Ran Chen, Zhong-Bing Wu, Jian-Yong Li, Ya-Qin Sun, Dong-Xuan Shi, Jing Li

**Affiliations:** ^1^ Department of Traditional Chinese Medicine, The Fourth Hospital of Hebei Medical University, Hebei Cancer Hospital, Shijiazhuang, China; ^2^ Institute for Biotechnology, St. John’s University, Queens, NY, United States; ^3^ Pharmacy Department, The Fourth Hospital of Hebei Medical University, Hebei Cancer Hospital, Shijiazhuang, China; ^4^ College of Integrated Traditional Chinese and Western Medicine, Hebei Medical University, Shijiazhuang, China; ^5^ Hebei Ping An Health Group Pharmaceutical Research Institute, Shijiazhuang, China; ^6^ Health Food Research Office, Shijiazhuang Yiling Pharmaceutical Co., Ltd., Shijiazhuang, China

**Keywords:** esophageal precancerous lesions, Siwu-Yin, synthesis and secretion of bile acids, macrophage polarization, metabolomics, traditional Chinese medicine

## Abstract

Siwu-Yin (SWY), a traditional Chinese medicinal formula, can replenish blood and nourish Yin. It was recorded in ancient Chinese medicine books in treating esophageal dysphagia, which has similar symptoms and prognosis with esophageal precancerous lesions and esophageal cancer. However, its effect has not been established *in vivo*. This study explores the antiesophageal cancer effect of SWY on rats with esophageal precancerous lesions. By performing 16S rRNA gene sequencing and metabolomics, it was suggested that SWY may improve the composition of intestinal flora of rats by regulating the synthesis and secretion of bile acids. In addition, flow cytometry results showed that SWY treatment modified tumor microenvironment by improving macrophage polarization and therefore inhibiting the occurrence of esophageal precancerous lesions.

## 1 Introduction

Traditional Chinese medicine (TCM) has several thousand years of history. It is mainly derived from nature, such as botanical drug medicine and insect medicine. In clinical practice, Chinese people often use a TCM formula with complex ingredients to treat diseases. Although TCM has a good reputation and extensive clinical application in modern Chinese society, the evaluation of its effects is often based on the self-perception of patients and lacks laboratory scientific certification or high-level clinical trials. Thanks to the advancement of scientific technology, it has become possible to verify the mechanism of a complex formula.

The incidence of esophageal cancer varied in regions. China is the country with the highest prevalence and mortality of esophageal cancer in the world (Bray et al., 2018). The region surrounding the Taihang Mountains is one of the areas that have the highest incidence of esophageal cancer in China (Totsuka et al., 2019). Through long-term clinical experience and thorough observations, the clinical doctors in our research group found that most patients with esophageal cancer had symptoms of dry mouth, constipation, and weight loss, and treatment using TCM for supplementing blood and nourishing Yin achieved remarkable results in the management of esophageal cancer (Shi et al., 2016; Kong et al., 2019). Siwu-Yin (SWY), which can replenish blood and nourish Yin, is a classic recipe for replenishing blood in TCM. The records from some famous TCM literature such as *The Orthodox Tradition of Medicine*, *Complete Works of Zhang Jingyue*, and *The Secrets of Treating Wounds and Bonesetting* show that the ingredients in SWY may cure refractory esophageal dysphagia, which has similar symptoms and prognosis to esophageal cancer (Yuanlin Zhang et al., 2020). We observed that SWY was able to improve the clinical symptoms of patients with esophageal precancerous lesions. While SWY has been shown to prevent esophageal precancerous lesions in clinical settings, fundamental evidence is lacking. Previous studies have shown that SWY can significantly inhibit the migration and invasion of esophageal cancer cells and can significantly improve the symptoms of dry mouth and constipation in patients with precancerous lesions of esophageal cancer (Shi et al., 2020). In the current study, we explore the antiesophageal cancer effect of SWY on esophageal precancerous lesions from the aspects of intestinal flora and serum metabolomics.

## 2 Materials and Methods

### 2.1 Siwu-Yin Lyophilized Powder

The components of SWY were purchased from Le Ren Tang Pharmacy in Shijiazhuang (Hebei, China) and identified by Dr. Xinran Chen (The Fourth Hospital of Hebei Medical University, Hebei, China). The SWY formula was prepared with *Angelica sinensis* (Oliv.) Diels., *Paeonia lactiflora* Pall., *Rehmannia glutinosa* (Gaertn.) DC., *Conioselinum anthriscoides* (H.Boissieu.), and *Clematis chinensis* Osbeck in a weight ratio of 1:1:2:1:1. A total of 60 g of botanical drug mixture was ground and steeped in 600 ml of pure water (1:10, w/v) for 30 min. The mixture was boiled for 1 h and the decoction was collected. Subsequently, the mixture was again steeped in 600 ml of pure water (1:10, w/v) for 30 min and boiled for 1 h. The decoction was collected and combined.

One hundred ten grams of *Zingiber officinale* Roscoe, 120 g of *Allium tuberosum* Rottler ex Spreng., 60 g of *Pyrus Bretschneider* Rehder, and 150 g of *Nelumbo nucifera* Gaertn. were rinsed and 10 ml of juice of each herb was collected using an extractor. Subsequently, milk [Mengniu Dairy (Group) Co., Ltd., Nei Menggu, China] and Succus Bambusae (MINJI Pharmaceutical Co., Ltd., Jiangxi, China) were added in a ratio of 1:1:1:1:6:1 (v/v) to a final volume of 110 ml. The extraction was combined with drug decoction in the ratio of 1:5 (v/v). Finally, the formula solution was pressure-filtered and centrifuged, and the supernatant was collected. Then, the supernatant was concentrated with a rotary evaporator, which yielded 30 g of dry SWY lyophilized powder and stored at −80°C.

### 2.2 Experimental Animals

F344 rats aged 4–5 weeks, with an equal number of males and females, SPF grade, weighing (200 ± 20) g, were provided by Beijing Weitong Lihua Laboratory Animal Technology Co. (Certificate No.: male rats: 1100111911076398, female rats: 1100111911076399). The purchased rats were kept in the animal experimental center. The constant temperature of the animal room is 23 ± 2°C and the relative humidity is 40%–60%, with 12-h daily illumination time. Animals were fed with standard animal feed, which is provided by the animal center of Hebei Medical University. The drinking water is pure water, which is sterilized at high temperature and changed every day.

### 2.3 Reagents and Instruments

Paeoniflorin (Desite Biotechnology Co., Ltd., China, Batch number: DS0071), Albiflorin (China Institute for Food and Drug Control, China, Batch number: 110736202044), Ferulic acid (China Institute for Food and Drug Control, China, Batch number: 110773-201915), Isoferulic acid (China Institute for Food and Drug Control, China, Batch number: 111698-201904), HiPure Stool DNA Kits (Magen, Guangzhou, China), VAHTSTM DNA Clean Beads (Vazyme Biotech, Nanjing, China), Chloral hydrate (Sinopharm Chemical Reagent, Shanghai, China, Batch number: 20141019), PE mark CD163 (BioRad, California, USA, Batch number: 154586), PE mark CD86 (BioRad, California, USA, Batch number: B292853), and FITC mark F480 (Biolegend, Beijing, China, Batch number: 152107).

Thermo Scientific Vanquish Core HPLC (Germany); Chromeleon 7 chromatography workstation; Chromatographic column: Waters Symmetry C18 (5 μm, 4.6 × 250 mm); Ag135 analytical balance (Mettler Toledo, Switzerland); KQ5200B ultrasonic cleaner (Kunshan Ultrasonic Instrument Factory, China). TD6001 digital balance (Sigma, United States); Ordinary slicers (Leica, Germany); The positive microscope (Leica, Germany); D3024R desktop high-speed frozen micro centrifuge (Scilogex, United States); CytoFLEX cytometry (Beckman, United States); FLx800 quantitative instrument Microplate reader (BioTek, United States); Ultra performance liquid chromatography (UPLC) for metabolome and intestinal flora analysis (ExionLC AD, https://sciex.com.cn/); and Tandem mass spectrometry (MS/MS) for metabolomics detection (QTRAP https://sciex.com.cn/).

### 2.4 Establishment of High-Performance Liquid Chromatographic Fingerprint

One gram of SWY powder was put into a conical flask with a stopper; 25 ml of 75% methanol was added and sonicated for 20 min. The solution was cooled and filtered with a 0.22-μM filter membrane (Li et al., 2008; Ye et al., 2017).

After previous experimental tests, the HPLC analytic conditions were determined as follows: Chromatographic column: Waters Symmetry C18 (5 μm, 4.6 × 250 mm). Mobile phase: A: acetonitrile, B: 0.1% phosphoric acid aqueous solution. Gradient: 0–20 min, 10–15% A; 20–30 min, 15–20% A; 30–38 min, 20–38% A; 38–40 min, 38–55% A; 40–45 min, 55–62% A; 45–50 min, 62% A; 50–60 min, 62–68% A. Detection wavelength: 280 nm. Column temperature: 35°C. Velocity of flow: 1.0 ml/min.

The retention time and UV spectrum of the reference substance were investigated through the reference substance test, and the chromatographic peaks of SWY in HPLC fingerprint were identified. The fingerprints of 10 batches of SWY were analyzed to obtain the chromatogram and their common mode.

### 2.5 Establishing the Rat Model of Esophageal Precancerous Lesions

A rat model of esophageal precancerous lesions induced by methylbenzylnitrosamine (MBNA) was established in F344 rats aged 4–5 weeks according to a previous study (Shi and Chen, 2014). Eight normal rats (4 males and 4 females) were used as the Blank group. Esophageal precancerous lesion model rats were randomly divided into the Model group and the Siwu group, with 16 rats in each group (8 males and 8 females). MBNA solution (0.30 mg/kg) was subcutaneously injected every 3 days. Rats in the Siwu group were given SWY in the drinking water from the beginning of the experiment.

According to the formula: 6.3 × mg/kg (x is the clinical dose of human, which is 35 g SWY for a normal individual of 70 kg), the dosage of rats was 3.15 g/kg/day. The water intake of rats was monitored, the concentration was calculated according to the body weight and water intake of rats, and SWY was diluted with distilled water to keep the intake relatively constant.

At 24, 32, and 40 weeks after modeling, two rats in the Model group and Siwu group were sacrificed, and esophageal hematoxylin and eosin (H&E) staining was used to observe the modeling. After 40 weeks of modeling, esophageal epithelium in the Model group showed atypical hyperplasia in varying degrees.

### 2.6 Sample Collection and Processing

After 40 weeks of modeling, the rats were weighed, and anesthetized by intraperitoneal injection of 10% chloral hydrate (0.35 ml/100 g). The blood from abdominal aorta was collected into the anticoagulant tube with heparin sodium and then subjected to centrifuge. The esophagus and spleen were collected, the weight of spleen was weighed, and the spleen index was calculated (Shi and Li, 2020). The intestinal contents were also collected. The plasma and the intestinal contents were stored at −80°C, buried in dry ice and transported to Guangzhou Bo Biotechnology Co., Ltd. to detect the intestinal flora.

### 2.7 H&E Staining

The esophageal tissue was fixed with 10% formalin and embedded in paraffin. Five sections were continuously sliced for H&E staining (Xiang et al., 2019). The histopathological changes of esophageal mucosa in each group were observed under a light microscope.

### 2.8 16S rRNA Gene Sequencing and Bioinformatics Analysis

Microbial DNA was extracted using the HiPure Stool DNA Kits (Magen, Guangzhou, China) from the contents of rat cecum and quantified. The 16S rDNA V3–V4 region of the ribosomal RNA gene was amplified by PCR (94°C for 2 min, followed by 30 cycles at 98°C for 10 s, 62°C for 30 s, and 68°C for 30 s and a final extension at 68°C for 5 min) using primers 341F: CCTAYGGGRBGCASCAG; 806R: GGACTACNNGGGTATCTAAT (Guo et al., 2017; Tong et al., 2018). Amplicons were purified using the VAHTSTM DNA Clean Beads (Vazyme Biotech, Nanjing, China) and quantified using the ABI Step One Plus Real-Time PCR System (Life Technologies, Foster City, USA). Purified amplicons were pooled in equimolar and paired-end sequenced (2 × 250) on an Illumina platform according to the standard protocols. The entire data were based on qiime2 analysis process. Sequence denoising is used to obtain the characteristic sequence, and the characteristic sequence is annotated to obtain the characteristic sequence table of all samples, and then α- and β-diversity analyses were performed. The differences between groups were compared to obtain the different bacteria between groups.

### 2.9 Non Targeted Metabolomics Detection of Plasma Metabolites

Sample was thawed on ice and mixed well, and 300 l of pure methanol was added to 50 l of blood samples. The samples were centrifuged with 12,000 rpm at 4°C for 10 min. Then, the supernatants were collected and centrifuged at 12,000 rpm at 4°C for 5 min. The supernatants were placed in a refrigerator at −20°C for 30 min, centrifuged at 12,000 r/min at 4°C for 3 min, and 150 l of supernatants was taken in the liner of the corresponding injection bottle for analysis (Zhao et al., 2018; Zheyu Zhang et al., 2020).

After the tests, the analysis conditions were determined as follows: The conditions of liquid phase: chromatographic column: Waters ACQUITY HPLC HSS T3 C18 (1.8 µm, 2.1 mm × 100 mm); Mobile phase: A: ultrapure water (0.1% formic acid), B: acetonitrile (0.1% formic acid); gradient program: 95:5 V/V at 0 min, 10:90 V/V at 10.0 min, 10:90 V/V at 11.0 min, 95:5 V/V at 11.1 min, 95:5 V/V at 14.0 min; column temperature: 40°C; injection volume: 2 μl.

The conditions of mass spectrometry are as follows: temperature 500°C; ion spray voltage (IS) 5500 V (positive), −4500 V (negative); ion source gas I (GSI), gas II (GSII), and curtain gas (CUR) were set at 55, 60, and 25.0 psi, respectively; the collision gas (CAD) was high. Instrument tuning and mass calibration were performed with 10 and 100 μmol/L polypropylene glycol solutions in QQQ and LIT modes, respectively. A specific set of MRM transitions was monitored for each period according to the metabolites eluted within the period. The mass spectrum data were processed by the Software Analyst 1.6.3.

### 2.10 Association Analysis Between 16S rRNA Gene Sequencing and Metabolomics

In order to integrate the data of the16S rRNA gene sequencing and metabolomics, orthogonal partial least squares discriminant analysis was performed (Witting, 2018; Sompairac et al., 2019). The data of the metabolite abundance were calculated by omics PLS in R language, and the O2PLS model was established. The Pearson correlation coefficient between the data of metabolomics and the data of microbiota at the phylum and family levels was calculated by R language. A heatmap was generated.

### 2.11 The M1 and M2 Macrophages Detected by Flow Cytometry

The spleen and the precancerous tissues of esophagus in rats were collected and washed with PBS to remove residual blood. The tissues were placed in an autoclaved mortar, ground with PBS, and passed through a 70-μM cell filter. Red blood cells were lysed on ice for 5 min. Samples were centrifuged at 4°C for 5 min (3500 r/min), washed, and centrifuged again. PBS was added to prepare a single-cell suspension, and the cell count was adjusted to 1 × 10^6^/ml (Xu et al., 2018).

M1 macrophages were labeled with F4/80 and CD86, and M2 macrophages were labeled with F4/80 and CD163. They were kept away from light at 4°C for 30 min, centrifuged at 4°C for 5 min (3500 r/min), and washed 2 times with PBS. After centrifugation, supernatant was discarded, and cells were fixed with paraformaldehyde and detected by flow cytometry (CytoFLEX) within 24 h (Xu et al., 2020). The results were processed and analyzed using the software CytExpert.

### 2.12 Statistical Analysis

The experimental data were statistically analyzed by SPSS 22.0. All experiments were repeated 3 times. The measurement data conformed to the normal distribution and were expressed as mean ± standard deviation (x̄ ± s). Two independent samples *t*-test was used for the comparison between the groups. The mean of multiple groups was compared by one-way ANOVA. Counting data were compared by *χ* ^2^ inspection. *p* < 0.05 was considered statistically significant.

## 3 Results

### 3.1 Establishment and Identification of SWY Fingerprint by HPLC

**FIGURE 1:**
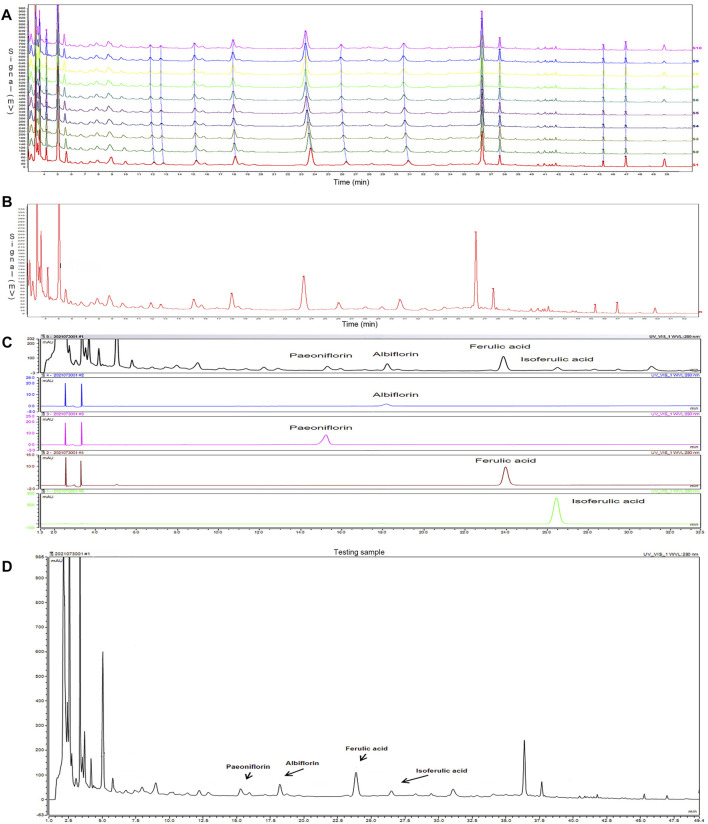
To establish the fingerprint of Siwu-Yin lyophilized powder (SWY) **(A)**, 10 batches of SWY **(B)** were analyzed by fingerprint, and the common pattern map of SWY was obtained. **(C)** The retention time and UV spectrum of SWY were compared with those of the control (Paeoniflorin, Albiflorin, Ferulic acid, and Isoferulic acid). **(D)** Some chromatographic peaks of SWY were identified through the reference substance test.

The fingerprint of SWY was established by HPLC: 10 batches of SWY were analyzed to obtain the chromatogram as shown in Figure 1A, and the common mode is shown in Figure 1B. The similarity of each sample with common mode was 0.988, 0.987, 0.985, 0.984, 0.999,0.999, 0.999, 0.999, 0.998, and 0.996, which was greater than 0.9. The results show that the ten batches of drugs have good similarity.

Positioning the chromatographic peak: the retention time and UV spectrum of the reference substance were investigated by the reference substance test, as shown in Figure 1C. Some HPLC fingerprint chromatographic peaks of SWY were identified, as shown in Figure 1D.

### 3.2 Effect of SWY on Rats With Esophageal Precancerous Lesions

**FIGURE 2:**
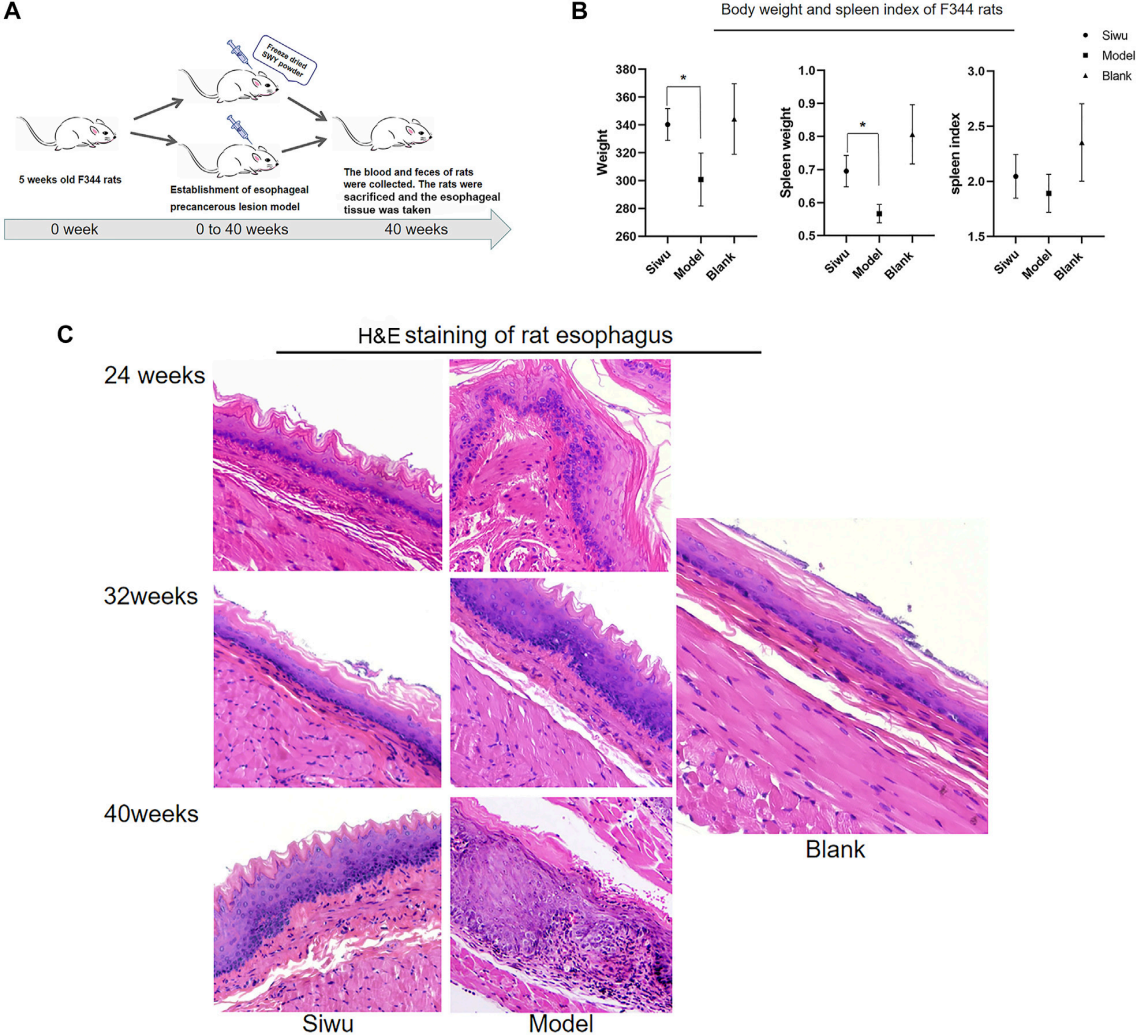
Effect of SWY on F344 rats with esophageal precancerous lesions. **(A)** The rat model of esophageal precancerous lesions was established by MBNA in F344 rats aged 4–5 weeks. The rats in each group were sacrificed after 40 weeks of NMBA intervention. **(B)** Effects of SWY on body weight and spleen index in rats with esophageal precancerous lesions. Compared with the Model group, the body weight and spleen weight of the Siwu group were significantly higher (all *p* < 0.05). The spleen index of the Siwu group was higher than that of the Model group, but the difference was not statistically significant (*p* > 0.05). **(C)** After 40 weeks of MBNA intervention, the esophageal epithelium of rats in the Model group showed atypical hyperplasia in varying degrees, and only epithelial hyperplasia appeared in the esophageal mucosa of rats in the Siwu group.

After the intervention with MBNA, as shown in Figure 2A, male rats were more successful than female rats in modeling, so the data of male rats were taken to complete the experiment. As shown in Figure 2B, the body weight and spleen weight of rats in the Siwu group were significantly higher than that of the Model group (*p* < 0.05). The spleen index of rats in the Siwu group was higher than that of the Model group, but without statistical significance (*p* > 0.05). The experimental results showed that after 40 weeks of MBNA intervention, the esophageal epithelium of rats in the Model group showed atypical hyperplasia in varying degrees, while only epithelial hyperplasia appeared in the esophageal mucosa of rats in the Siwu group, as shown in Figure 2C. These results indicated that SWY can inhibit precancerous lesions of esophagus in rats.

### 3.3 Effect of SWY on the Structure of Intestinal Flora in Rats With Esophageal Precancerous Lesions

#### 3.3.1 α-Diversity

**FIGURE 3:**
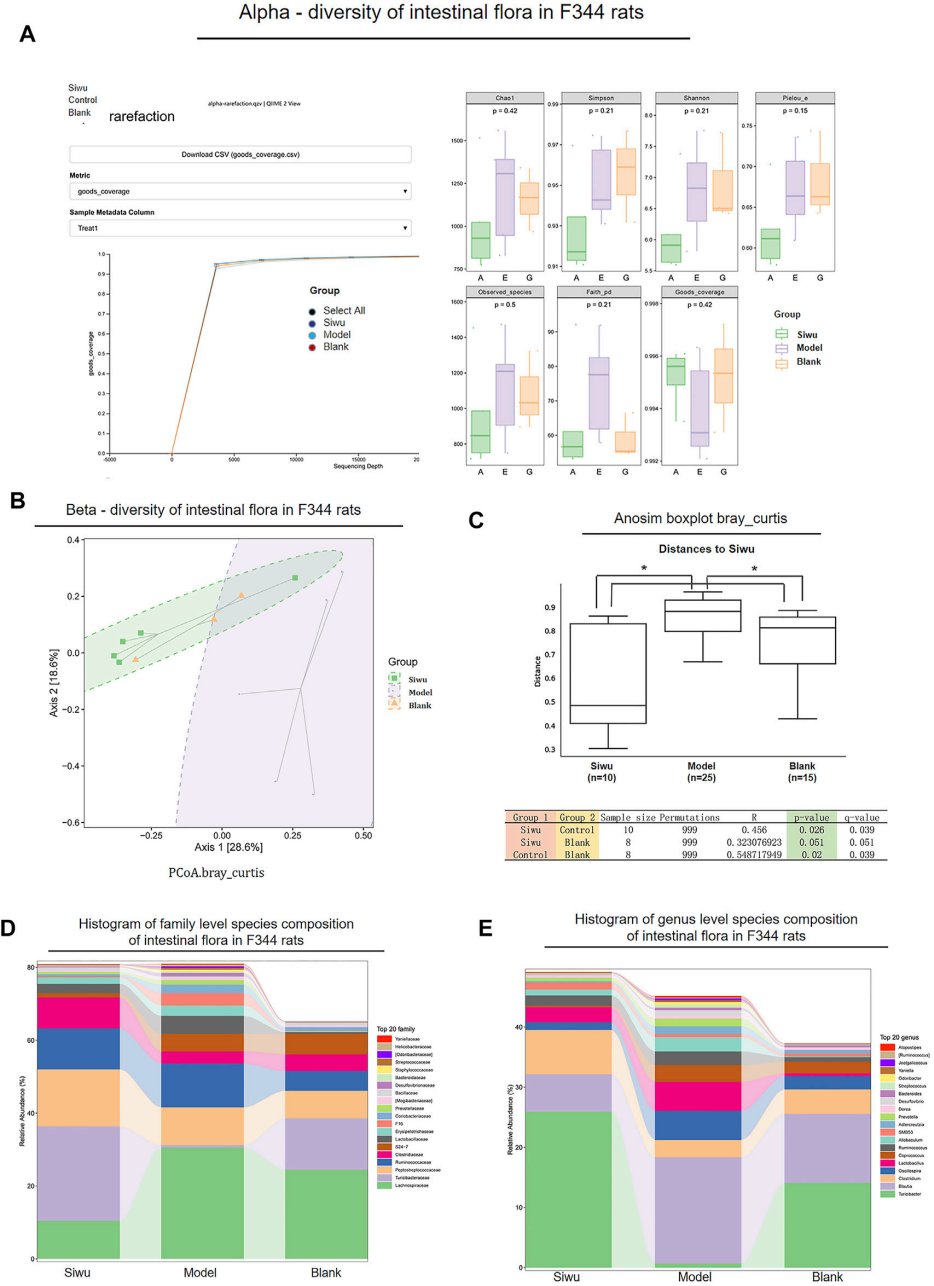
Effect of SWY on the intestinal flora community of rats with esophageal precancerous lesions. **(A)** In α-diversity, the α-Sparse curves of the three groups basically reach the platform period. The values of Goods_coverage, Chao1, observed_species, Shannon, and Simpson among the three groups were *p* > 0.05. **(B)** In β-diversity, the results of PCoA analysis showed that the sample distance between the Siwu group and the Blank group was similar, and the distance between the Model group and the other two groups was far. **(C)** In β-diversity, Anosim (analysis of similarities) analysis showed that the overall difference between the Siwu group and the Blank group was small, which had no statistical significance, and the overall difference between the Model group and the other two groups was significant. **(D)** The expression of the top 20 species in the three groups at the genus level. **(E)** The expression of the top 20 species in the three groups at the family level.

As shown in Figure 3A, the α-sparse curves of the Siwu group, Model group, and Blank group basically reached the plateau stage. Therefore, the sequencing results were sufficient to reflect the diversity of the tested samples, which is a prerequisite for comparing α-diversity.

The change of Goods_coverage showed no significant difference among these three groups (*p* > 0.05). The changes of Chao1, observed species, Shannon, and Simpson were not statistically significant among these three groups (*p* > 0.05). Therefore, there was no significant difference in species abundance and evenness among the samples of the three groups.

#### 3.3.2 β-Diversity

As shown in Figure 3B, the PCoA analysis results showed that the distance of the samples between the Siwu group and Blank group was close. It indicated that there was little difference in the number and distribution of floral species between the Siwu group and the Blank group. The distance of samples between the Model group and the other two groups was far, suggesting a great difference in the number and distribution of species between the Model group and the other two groups.

As shown in Figure 3C, the analysis of Anosim (analysis of similarities) showed that the difference between the Siwu group and the Blank group was not statistically significant, while the difference between the Model group and the other two groups was significant (*p* < 0.05).

#### 3.3.3 Species Composition

The expression of the top 20 species at the levels of family and genus in the samples of these three groups is shown in Figures 3D,E. At the family level, the abundance of Turicibacteraceae in the Model group was significantly lower than that in the Blank group. The abundance of Turicibacteraceae in the Siwu group was significantly higher than that in the Model group. At the genus level, the abundance of Turicibacter in the Model group was significantly lower than that in the Blank group, and the abundance of Turicibacter in the Siwu group was significantly higher than that in the Model group.

#### 3.3.4 Analysis of Iconic Species

Through the analysis of Iconic species, we attempted to find the ASV/OTU with significant difference among the samples of the three groups.

**FIGURE 4:**
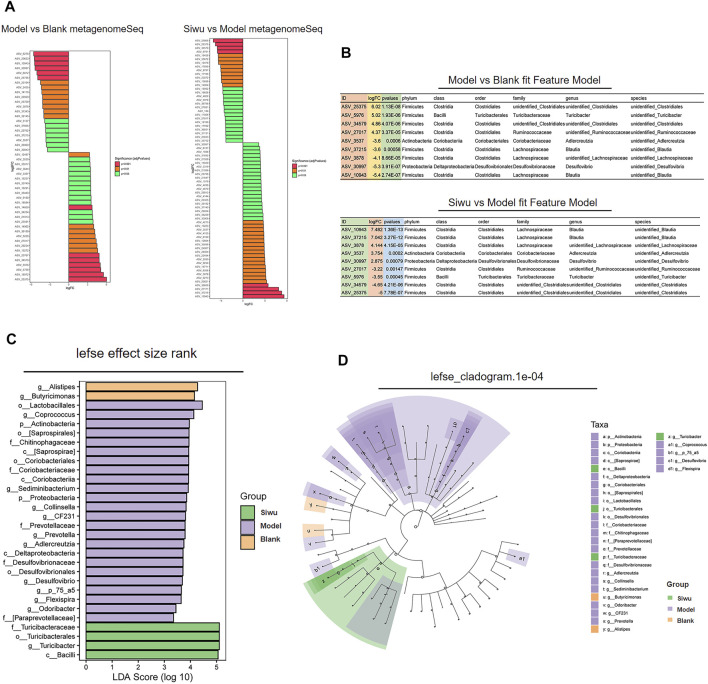
Analysis of marker species in intestinal flora. **(A)** The ASV/OTU with the statistically significant difference between the Model group and the Blank group and between the Siwu group and the Model group. **(B)** The ASV/out, which significantly changed after modeling and corrected after SWY intervention from the two sets of data, and the log2 value of ASV/OTU multiples are shown. **(C)** The marker metabolites of each group are shown. **(D)** The taxonomic distribution map shows the taxonomic–hierarchical relationships from phylum to genus in the sample of each group.

We evaluated whether these different ASVs/OTUs had an enrichment trend at different classification levels. Figure 4A shows the ASV/OTU with statistically significant differences between the Model group and the Blank group and the ASV/OTU with statistically significant differences between the Siwu group and the Model group. The ASV/OTU was found to be changed after modeling and then corrected after SWY treatment from the two data. The results are shown in Figure 4B. In the log2 of ASV/OTU (fold change; FC) between two groups, a positive value meant that the expression was upregulated and a negative value meant downregulation of expression. Figure 4C shows the marker species of intestinal flora in each group. Figure 4D shows the taxonomic hierarchy from phyla to genus in the sample of each group. At the levels of class, order, family, and genus, the marker species of the Siwu group are as follows: c_ Bacilli; o_ Turicibacterales; f_ Turicibacteraceae; g_ Turicibacter.

The results show that the enrichment of Turicibacteraceae and Turicibacter in intestinal flora of esophageal precancerous lesion model rats was significantly lower than that of normal rats, and SWY could increase the abundance of Turicibacteraceae and Turicibacter.

### 3.4 Effect of SWY on Plasma Metabolites in Rats With Esophageal Precancerous Lesions

In order to understand whether SWY could improve the metabolism of rats *via* improving the enrichment of Turicibacteraceae and Turicibacter, we further analyzed the metabolites in rat plasma. The plasma samples were stored in a refrigerator at 4°C, and the repeatability test results showed that the GC-MS instrument had good repeatability (relative standard deviation RSD <5.0%).

#### 3.4.1 Plasma Analysis

**FIGURE 5:**
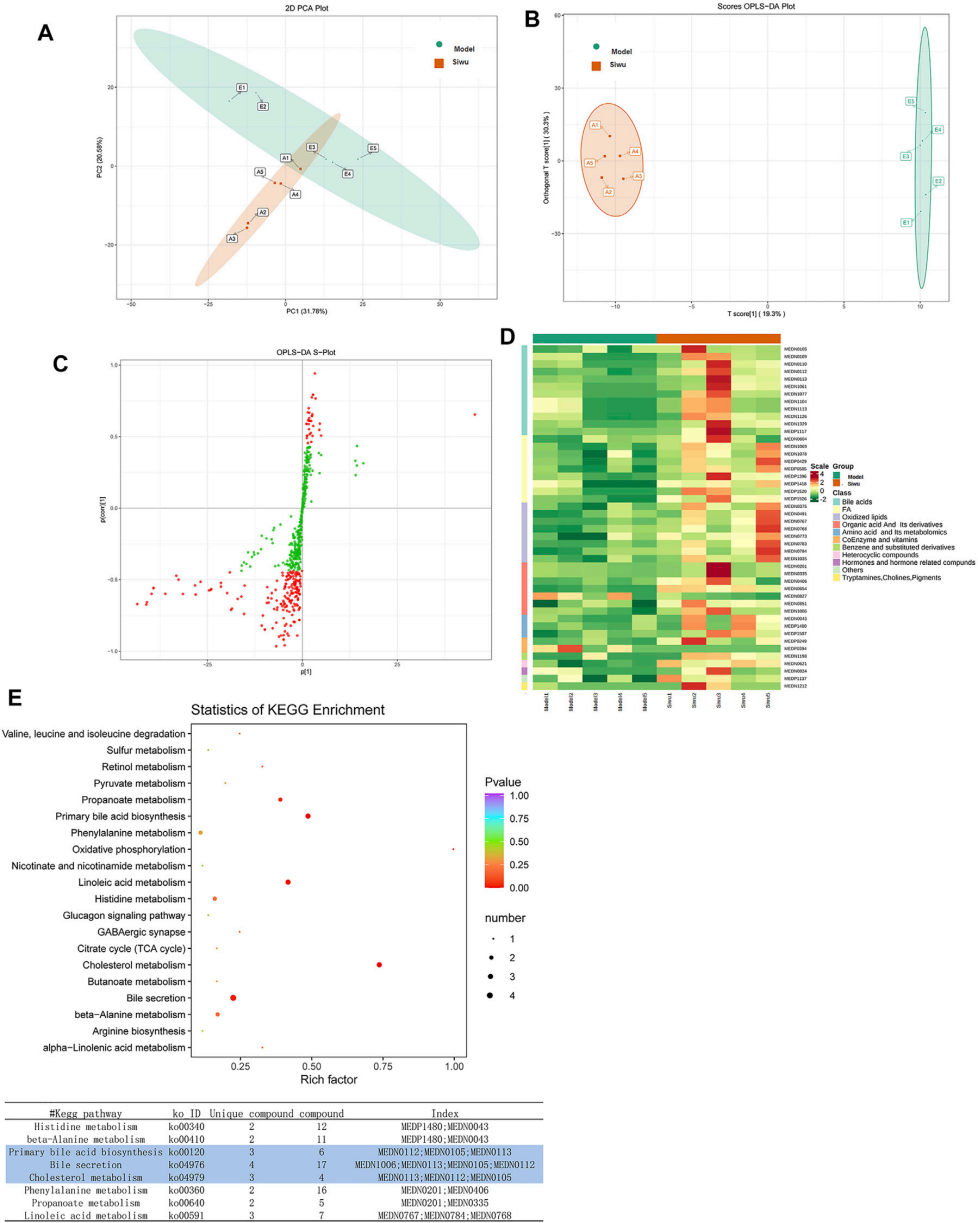
Effect of SWY on the metabolites of plasma in rats with esophageal precancerous lesions. **(A)** The PCA method was used to investigate the dispersion degree of the Siwu group and the Model group. **(B)** In order to find the differential metabolites related to the intervention of SWY, OPLS-DA analysis was carried out between the Siwu group and the Model group. The OPLS-DA score chart shows that the Siwu group was completely separated from the Model group. **(C)** In the OPLS-DA S-plot, the VIP value of metabolites marked with red dots was greater than or equal to 1, which made a relatively large contribution to the screening of differential metabolites. **(D)** Based on the OPLS-DA results, select the metabolites with VIP ≥1, normalize the significantly different metabolites, and draw the cluster heatmap. **(E)** The differential metabolites were analyzed by KEGG database to clarify the pathways that were formed by the interaction of the differential metabolites *in vivo*.

The PCA method was used to investigate the dispersion degree of metabolites of rats in the Siwu group and the Model group. The results showed that there was a separation trend between the two groups, but there was partial overlap and did not completely distinguish, as shown in Figure 5A. In order to find the differential metabolites in plasma of rats treated by SWY, OPLS-DA analysis was carried out between the Siwu group and the Model group. The OPLS-DA score chart is shown in Figure 5B, and the OPLS-DA S-plot is shown in Figure 5C. As shown in the OPLS-DA score chart, the Siwu group was completely separated from the Model group, which indicated that the metabolic status of the two groups was significantly different. This result showed that SWY significantly changed the blood metabolites of the model rats. In the OPLS-DA S-plot, the VIP value of the red point is greater than or equal to 1, indicating a relatively large contribution. We evaluated the differential metabolites by observing the variables with VIP greater than or equal to 1.

#### 3.4.2 Identification of Differential Metabolites

**FIGURE 6:**
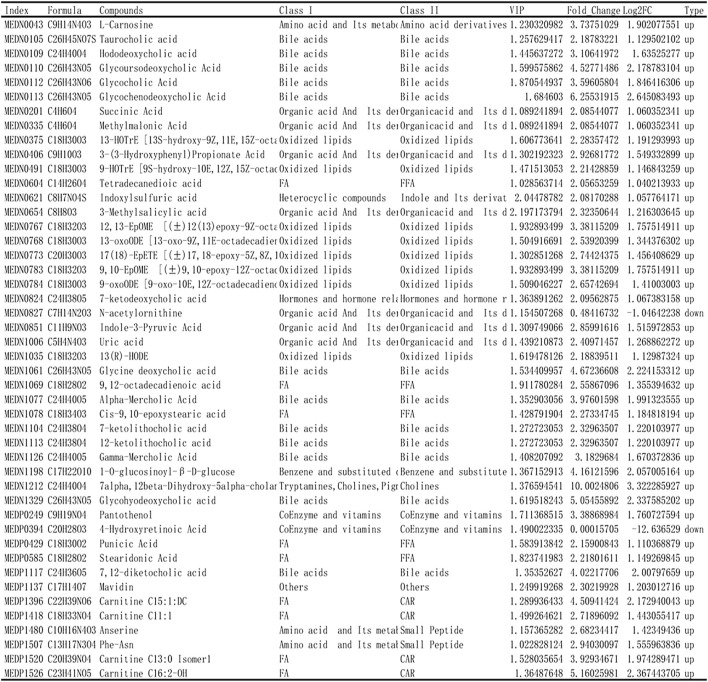
Identification of differential metabolites. Based on the results of OPLS-DA, the metabolites with VIP ≥ 1 were selected and combined with the fold change to further screen the differential metabolites.

Based on the results of OPLS-DA, the metabolites with VIP ≥1 were selected. The differential metabolites between the two groups were preliminarily screened, and then the differential metabolites were further screened with the fold change of the value, as shown in Figure 6. In order to observe the change, a cluster heatmap was drawn (Figure 5D). The metabolites with significant differences were mostly concentrated in bile acids, free fatty acids, and oxidized lipids.

#### 3.4.3 The Metabolic Pathways Affected by SWY

Differential metabolites were analyzed using the KEGG database to identify the pathway of differential metabolites *in vivo*. The results are shown in Figure 5E. A lower *p* value indicates a more significant enrichment. Rich factor is the ratio of the number of the differential metabolites to the total number of metabolites in the pathway. The greater the value, the greater the enrichment degree. The results showed that before and after SWY treatment, the three metabolic pathways changed most significantly as follows: bile secretion, primary bile acid biosynthesis, and cholesterol metabolism.

These results suggested that there were significant changes in blood metabolites of rats with esophageal precancerous lesions as shown in the metabolomics before and after SWY treatment. After SWY treatment, the metabolic pathway change was related to bile acid synthesis and secretion.

### 3.5 Association Analysis of 16S rRNA Gene Sequencing and Metabolomics

**FIGURE 7:**
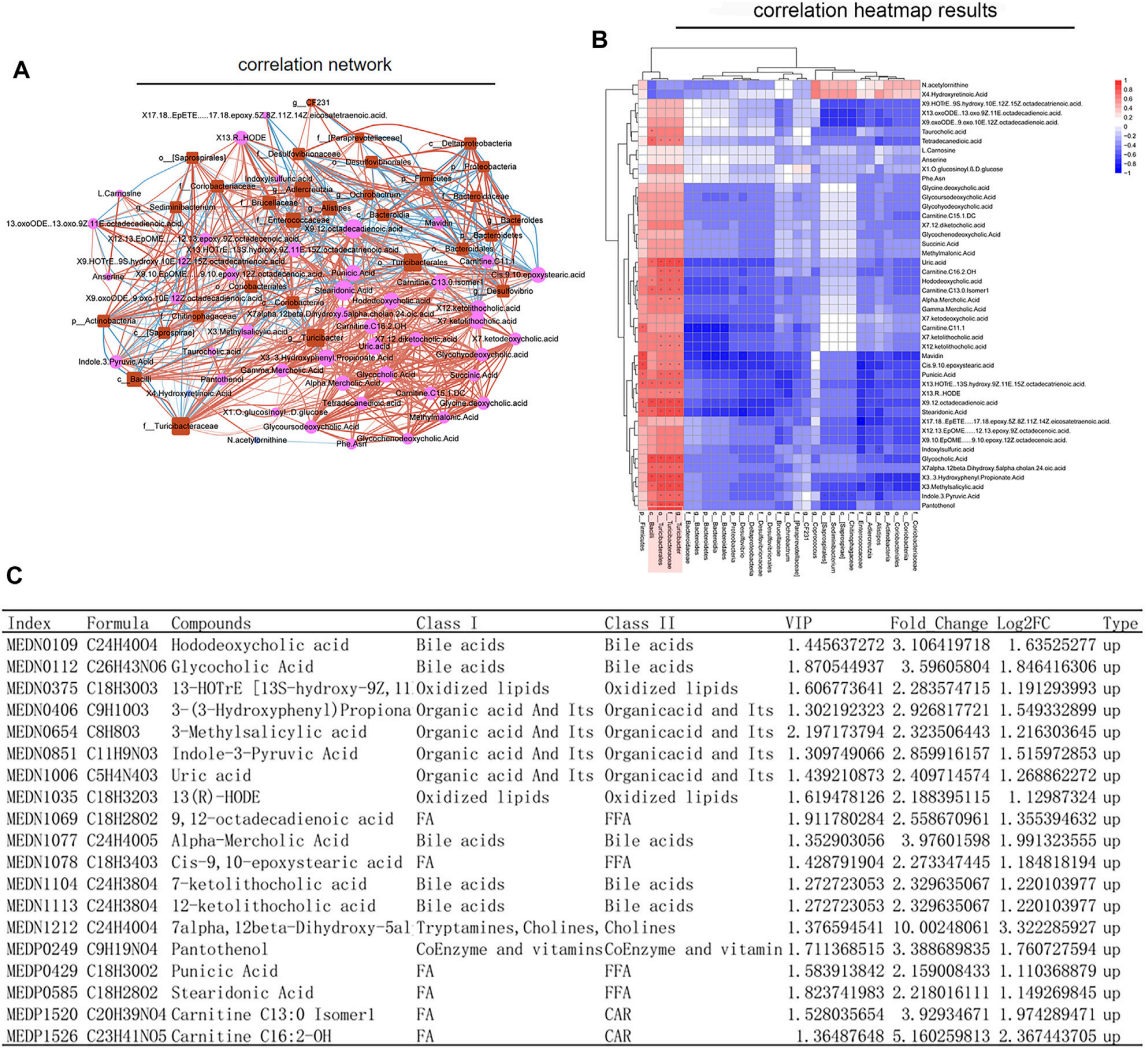
Combined analysis of intestinal flora and metabolism. **(A)** According to the network diagram of the combined analysis, the changes in intestinal flora could cause complex changes in the metabolites. **(B)** According to the heatmap of the combined analysis, the changes in the expression of metabolites are caused by the changes in intestinal flora. **(C)** The table listed the changes in plasma metabolites caused by the changes in Bacilli, Turicibacterales, Turicibacteraceae, and Turicibacter.

In order to understand whether the above results related to the changes of intestinal flora in rats before and after SWY intervention, we further made a combined analysis of intestinal flora and metabolites to clarify the correlation between them. As shown in Figure 7A, changes in intestinal flora could cause complex changes in plasma metabolites. The changes in intestinal flora were closely related to the changes in metabolites, as shown in Figure 7B. In the intestinal flora of rats with esophageal precancerous lesions, the expression of the outline Bacilli, the genus *Turicibacter*, the family turicibacteae, and the order turicibacterales decreased. SWY could improve their abundance, and the result was closely related to the changes of metabolites such as bile acids and fatty acids, as shown in Figure 7C. The results of metabolome showed that after the intervention of SWY, the main changed metabolic pathways were related to bile acid synthesis and secretion.

### 3.6 Effect of SWY on Macrophage Polarization in Rats With Esophageal Precancerous Lesions

It was not known whether SWY inhibits the occurrence of esophageal precancerous lesions *via* regulating the synthesis and secretion of bile acids. Bile acid is a special kind of steroids, and many studies have shown that bile acid is closely related to macrophage polarization (Cipriani et al., 2011; Deutschmann et al., 2018; Hegyi et al., 2018; Kemis et al., 2019). In order to understand whether SWY affected the macrophage polarization by regulating the synthesis and secretion of bile acid, we used flow cytometry to detect the effect of SWY on macrophage polarization in rats with esophageal precancerous lesions.

We used flow cytometry to observe the expression of M1 macrophages and M2 macrophages in splenocytes and esophageal precancerous lesions of rats. M1 macrophages were labeled with F4/80 and CD86, and M2 macrophages were labeled with F4/80 and CD163.

**FIGURE 8:**
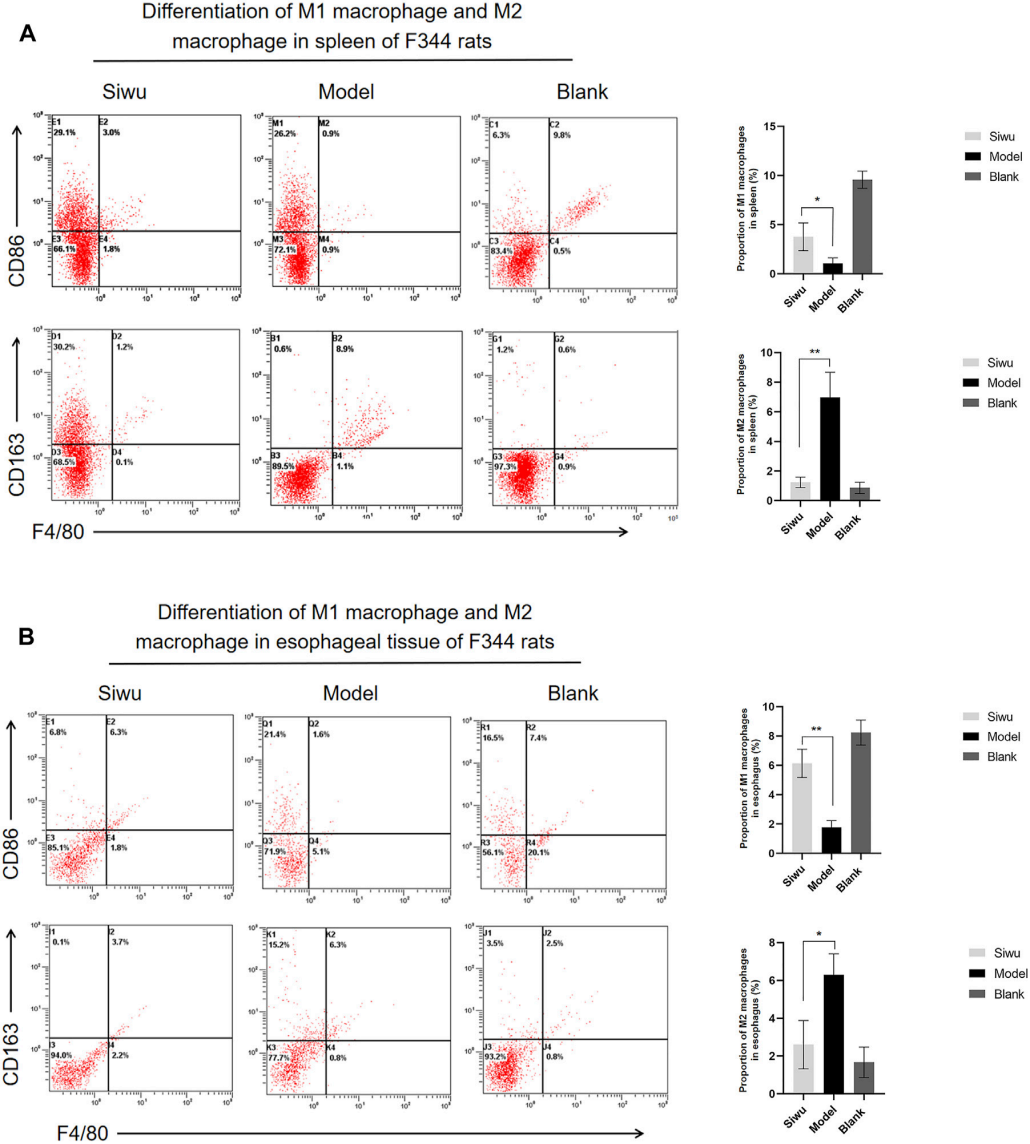
Effect of SWY on macrophage polarization in rats with esophageal precancerous lesions. **(A)** The proportion of M1 macrophages and M2 macrophages in spleen tissue of F344 rats was observed by flow cytometry. **(B)** The proportion of M1 macrophages and M2 macrophages in the esophagus of F344 rats was observed by flow cytometry.

In the rat splenocytes, the expression of M1 macrophages was significantly higher, while the expression of M2 macrophages was significantly lower in rats in the Siwu group than those in Model group (*p* < 0.005). The results are shown in Figure 8A. The expression of M1 and M2 macrophages in esophageal precancerous lesions of rats was consistent with that in rat splenocytes (*p* < 0.005), and the results are shown in Figure 8B. The results showed that SWY could improve the polarization of macrophages in rats with esophageal precancerous lesions.

## 4 Discussion

SWY, as a TCM formula, has been used to treat esophageal precancerous lesions mainly based on ancient Chinese medical literature. According to the records, the composition of SWY has the effect of treating clinical refractory dysphagia, which shares similar symptoms and prognosis of esophageal cancer (Shi, 2020). The obstacle of TCM formulas is that although they have optimal effects in clinical usage, the underlying mechanisms of action are inconclusive (Sheridan et al., 2012; Chen et al., 2021a; Chen et al., 2021b). Previous studies have shown that SWY can significantly improve the symptoms of dry mouth and constipation in patients with esophageal precancerous lesions. In addition, SWY has the effect of inhibiting the migration and invasion of esophageal cancer cells (Shi et al., 2020). Therefore, we conducted this study to evaluate the effect of SWY on intestinal flora and serum metabolomics in esophageal precancerous lesions.

In this study, MBNA was used to establish a rat model of esophageal precancerous lesions, and SWY was used for the treatment from the beginning of modeling. Interestingly, compared to female rats, male rats were more susceptible to the intervention of MBNA. Such phenomenon was not observed in other studies, which deserves future investigation (Schneider et al., 1996; Zanesi et al., 2001; Fong et al., 2003; Zhu et al., 2020). The experimental results confirmed that after 40 weeks of MBNA intervention, the esophageal epithelium of rats in the Model group showed atypical hyperplasia in varying degrees, and only epithelial hyperplasia appeared in the esophageal mucosa of rats in the Siwu group. Compared with the Model group, the body weight and spleen weight of rats in the Siwu group were significantly higher. These results confirmed that SWY could inhibit the occurrence of esophageal precancerous lesions. Through the analysis of rat intestinal flora, it was found that the abundance of turicibacter in the intestinal flora of rats with esophageal precancerous lesions decreased, and SWY could improve its abundance. The abundance of turicibacter was closely related to the metabolic pathway of bile acid synthesis and secretion (Kemis et al., 2019). The expression of M1 macrophages and M2 macrophages in rat splenocytes and esophageal precancerous lesions was detected by flow cytometry. M1 macrophages can present antigen, initiate Th1 immune response, and kill foreign antigens including tumor cells, and therefore inhibit tumorigenesis. M2 macrophages cause Th2 response; promote angiogenesis; destroy cell basement membrane; cause the proliferation, metastasis, and invasion of tumor cells; and finally promote tumor progression (Lin et al., 2016; Chen et al., 2018). The results showed that the number of M1 macrophages in the Model group was significantly lower than that in the Siwu group, and the number of M2 macrophages was significantly higher than that in Siwu group, indicating that SWY could improve the polarization of macrophages in rats with esophageal precancerous lesions.

Our study confirmed that SWY can improve macrophage polarization, which may be achieved by regulating the synthesis and secretion of bile acids *via* improving the intestinal flora of rats with esophageal precancerous lesions. Bile acids are mainly synthesized in the liver and secreted into the intestine. They not only promote the digestion and absorption of lipids, but also play an important role in physiological signals and metabolic regulation and regulate the homeostasis of the immune system (Testa et al., 2017). The effect of bile acids on the occurrence and development of esophageal cancer may be closely related to the concentration of bile acids (Souza et al., 2008; Mermelstein et al., 2018). As ligands, bile acids can activate a variety of receptors. Physiological doses of bile acids can regulate immunity by binding to receptors such as FXR and TGR5 (Jadhav et al., 2018). On the one hand, receptors TGR5 and FXR are expressed in macrophages. In the macrophages of human and rodent, the activation of these receptors can effectively inhibit the proinflammatory activity of macrophages (Chen et al., 2018). On the other hand, bile acids can induce the TGR5 signaling pathway of macrophages, which plays a pro-inflammatory or anti-inflammatory role by activating TGR5 on the surface of M1 macrophages and M2 macrophages, respectively (Jia et al., 2018). However, the relationship between bile acid and the polarization of macrophages warrants further study.

Clinically, the TCM formula prescribed for patients has fixed decoction requirements, such as decoction time and the amount of solvent (water). As a TCM formula, the composition of SWY is established by our group based on ancient Chinese medicine books and long-term clinical practice. We observed that SWY was able to improve the clinical symptoms of patients with esophageal precancerous lesions. While SWY has been shown to prevent esophageal precancerous lesions in clinical settings, fundamental evidence is lacking. Hence, we conducted this study to explore the potential mechanisms. Since this formula has not been tested in any animal model, we used clinical recommended dosage to calculate that for rat treatment to mimic the real-life situation. Combined with our previous *in vitro* study (Shi, 2020), the dose we selected is shown to be safe and reachable in humans. One limitation of this study is that, as a single-dose study, investigating the effect of different doses may provide more information for clinical practice. Another limitation is that there are certain drawbacks in our experiments using intraperitoneal injection of chloral hydrate for anesthesia. Chlorohydrate is not suitable for small animal experiments because it is considered to have only hypnotic effects, and intraperitoneal injection will cause adverse effects such as peritonitis and gastric ulcers in experimental animals. Since the experiment in this paper is not a survival surgery, and the concentration of chloral hydrate we use is low, the risk of adverse reactions caused by chlorohydrate can be avoided to a certain extent. Although some researchers suggest that chlorohydrate can produce deep anesthesia and analgesia (Rodrigues et al., 2006), the potential suffering that chlorohydrate may cause to laboratory animals cannot be ignored. In terms of pharmacological effects, an article indicated that including chloral hydrate and other commonly used anesthetics, such as isoflurane, halothane, ketamine/xylazine, chloral hydrate, and propofol anesthesia, will affect blood parameters (Schwarzkopf et al., 2013). In our study, all three groups received chloral hydrate injections; therefore, it may not affect the metabolites we identified and studied. In addition, the side effects of chloral hydrate on protein and mRNA levels were not the objective of our experiments. In future studies, we will focus on optimizing the dose of SWY formula and the ratio of each botanical drug. Since chloral hydrate may have some limitations in small animal studies, it is necessary to add anesthetic control experiments to select a more optimal anesthesia protocol.

Taken together, this study confirmed that SWY could inhibit the occurrence of esophageal precancerous lesions and improve the polarization of M1 macrophages.

## Data Availability

The original contributions presented in the study are included in the article/Supplementary Material, further inquiries can be directed to the corresponding author.
