# Supplementary material for: Chinese Medicine Formula Siwu-Yin Inhibits Esophageal Precancerous Lesions by Improving Intestinal Flora and Macrophage Polarization
Source: Front Pharmacol. 2022 Mar 3;13:812386. doi: 10.3389/fphar.2022.812386 (PMC8927885; doi:10.3389/fphar.2022.812386)
Supplement: Supplementary file 1 [file DataSheet1.DOCX]

<https://www.jianguoyun.com/p/DV1YpmIQucOBChjxpp4E>    （Combined analysis of metabolome and intestinal flora）

<https://www.jianguoyun.com/p/DVlZVwYQucOBChjTvJwE>     （Flow cytometry, in which LMD format has been supplemented）

<https://www.jianguoyun.com/p/DSwVwX8QucOBChimp54E>     （Streaming data）

<https://www.jianguoyun.com/p/DZ6tVMcQucOBChjQvJwE>          (Results of serum metabolome in rats)

<https://www.jianguoyun.com/p/DURliUEQucOBChjSvJwE>           (HE staining)

<https://www.jianguoyun.com/p/DVlZVwYQucOBChjTvJwE>          (Flow cytometry)

<https://www.jianguoyun.com/p/DaFb4QgQucOBChjWvJwE>       （Combined analysis of metabolome and intestinal flora）

<https://www.jianguoyun.com/p/DbsX_SMQucOBChjXvJwE>        （16s）

<https://www.jianguoyun.com/p/DTehOFkQucOBChjZvJwE>            (Weight)

<https://www.jianguoyun.com/p/DQUsS4AQucOBChjo45sE>           (HPLC-PDA)
